# Supplementary material for: KorB switching from DNA-sliding clamp to repressor mediates long-range gene silencing in a multi-drug resistance plasmid
Source: Nat Microbiol. 2025 Jan 23;10(2):448–67. doi: 10.1038/s41564-024-01915-3 (PMC11790492; doi:10.1038/s41564-024-01915-3)
Supplement: Supplementary file 1 — Supplementary Discussion and figure. [file 41564_2024_1915_MOESM1_ESM.pdf]

nature microbiology

Supplementary information https://doi.org/10.1038/s41564-024-01915-3

KorB switching from DNA-sliding clamp to repressor mediates long-range gene silencing in a multi-drug resistance plasmid

In the format provided by the authors and unedited

Supplementary Discussion

Structural alignment of the tripartite complex vs. the CTPɣS-bound KorBΔN30ΔCTD complex showed a low root mean square displacement value (RMSD), suggesting that KorBΔN30ΔCTD in the tripartite complex has already adopted an NTD-engaged conformation, even though CTPɣS was not added to the crystallization setup. A closer inspection of KorB NTD in the tripartite complex revealed that helices α3 and α4 from each subunit bundle together (bundling-in conformation) (see attached Figure), in contrast to the CTPɣS-bound KorBΔN30ΔCTD structure in which helix α3 swings outwards to pack against α4' from the opposing subunit (swinging-out conformation) (Figure a). The reciprocal exchange of helices in the swinging-out conformation maintained packing at the α3-α4 protein core of the KorB NTD and was likely driven by CTPɣS-binding (Figure a). The bundling-in conformation of α3-4 is often associated with the NTD-disengaged open-clamp conformation of ParB/ParB-like proteins[1–5], while the swinging-out conformation is often observed for a nucleotide-bound NTD-engaged closed-clamp conformation[1,6,7]. For canonical ParB, the closed-clamp conformation was demonstrated to be energetically favorable but the transition to the closed-clamp state is slow without CTP and cognate DNA[6,8]. Therefore, we reasoned that KorA-DNA may facilitate this transition or capture KorBΔN30ΔCTD in the NTD-engaged state, as observed in the tripartite structure. The two opposing *OB* DNA-binding domains (DBD) of KorB are also closer together when bound to KorA (inter-domain distance = ~30 Å) than when they are bound to *OB* DNA alone (inter-domain distance = ~34 Å; PDB: 1R71)[9] (Figure b), which is incompatible with specific binding to the *OB* site and suggests that KorA binding stabilizes a closed-clamp conformation that is more compatible with sliding on DNA.

1. Jalal, A. S. *et al.* A CTP-dependent gating mechanism enables ParB spreading on DNA. *Elife* **10**, e69676 (2021).
2. Jalal, A. S. *et al.* CTP regulates membrane-binding activity of the nucleoid occlusion protein Noc. *Mol Cell* S1097-2765(21)00505–0 (2021) doi:10.1016/j.molcel.2021.06.025.
3. Leonard, T. A., Butler, P. J. G. & Löwe, J. Structural analysis of the chromosome segregation protein Spo0J from Thermus thermophilus. *Mol. Microbiol.* **53**, 419–432 (2004).
4. Chen, B.-W., Lin, M.-H., Chu, C.-H., Hsu, C.-E. & Sun, Y.-J. Insights into ParB spreading from the complex structure of Spo0J and parS. *Proc. Natl. Acad. Sci. U.S.A.* **112**, 6613–6618 (2015).
5. Sukhoverkov, K. V. *et al.* The CTP-binding domain is disengaged from the DNA-binding domain in a cocrystal structure of Bacillus subtilis Noc-DNA complex. *J Biol Chem* **299**, 103063 (2023).
6. Soh, Y.-M. *et al.* Self-organization of parS centromeres by the ParB CTP hydrolase. *Science* **366**, 1129–1133 (2019).
7. Osorio-Valeriano, M. *et al.* The CTPase activity of ParB determines the size and dynamics of prokaryotic DNA partition complexes. *Mol Cell* **81**, 3992-4007.e10 (2021).
8. Antar, H. *et al.* Relief of ParB autoinhibition by parS DNA catalysis and recycling of ParB by CTP hydrolysis promote bacterial centromere assembly. *Sci Adv* **7**, eabj2854 (2021).
9. Khare, D., Ziegelin, G., Lanka, E. & Heinemann, U. Sequence-specific DNA binding determined by contacts outside the helix-turn-helix motif of the ParB homolog KorB. *Nat. Struct. Mol. Biol.* **11**, 656–663 (2004).

Supplementary Figure

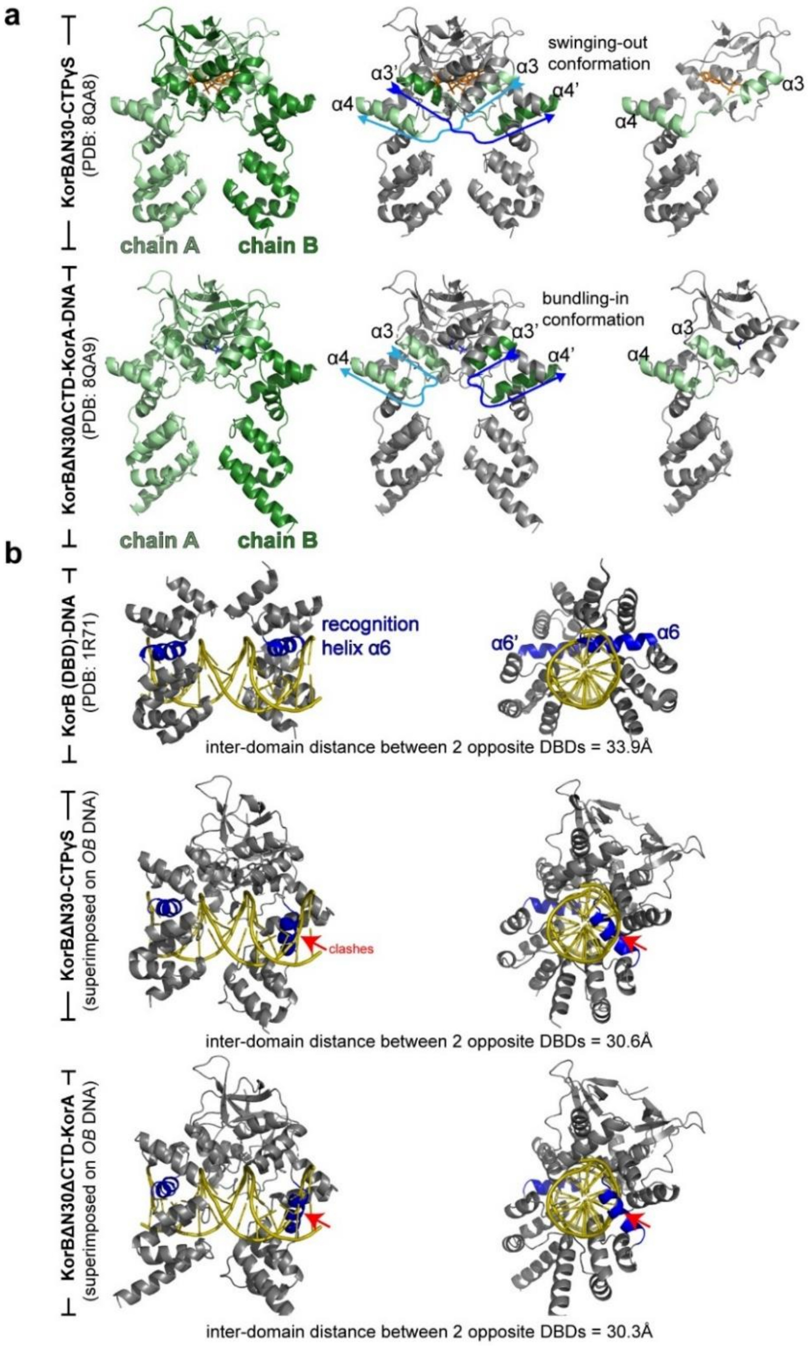

KorB adopts a closed-clamp conformation in the co-crystal structures of KorBΔN30ΔCTD-CTPγS complex and KorBΔN30ΔCTD-KorA-DNA complex, and the closed-clamp conformation is incompatible with site-specific DNA-binding at the *OB* site. a, Structures of closed-clamp KorBΔN30ΔCTD dimers with the pair of helices α3 and α4 colored differently (light green and dark green) to highlight the "swinging-out" or the "bundling-in" conformations. Light blue and dark blue arrows indicate the direction of the α3-α4 pair or the α3'-α4' pair from the opposite subunit. **b** The structure of a nucleotide-bound or KorA-bound KorBΔN30ΔCTD is incompatible with specific *OB* binding at the DNA-binding domain (DBD). Superimposing the KorBΔN30ΔCTD-CTPγS and KorBΔN30ΔCTD-KorA-DNA structures onto *OB* DNA (from the previously published KorB(DBD only)-*OB* co-crystal structure, PDB: 1R71) shows DNA-recognition helices (α6 and α6′, dark blue) positioning closer and away from the two consecutive major grooves of *OB* DNA (yellow), and helices α6 and α6′–α9′ at the DBD (red arrows) clashing with *OB* DNA.
