# Supplementary material for: KorB switching from DNA-sliding clamp to repressor mediates long-range gene silencing in a multi-drug resistance plasmid
Source: Nat Microbiol. 2025 Jan 23;10(2):448–67. doi: 10.1038/s41564-024-01915-3 (PMC11790492; doi:10.1038/s41564-024-01915-3)
Supplement: Supplementary file 2 — Reporting Summary [file 41564_2024_1915_MOESM2_ESM.pdf]

nature portfolio

Corresponding author(s): Tung Le

Last updated by author(s): 23/11/2024

Reporting Summary

Nature Portfolio wishes to improve the reproducibility of the work that we publish. This form provides structure for consistency and transparency in reporting. For further information on Nature Portfolio policies, see our Editorial Policies and the Editorial Policy Checklist.

Statistics

For all statistical analyses, confirm that the following items are present in the figure legend, table legend, main text, or Methods section.

n/a	Confirmed	
☐	☒	The exact sample size (*n*) for each experimental group/condition, given as a discrete number and unit of measurement
☐	☒	A statement on whether measurements were taken from distinct samples or whether the same sample was measured repeatedly
☐	☒	The statistical test(s) used AND whether they are one- or two-sided<br>*Only common tests should be described solely by name; describe more complex techniques in the Methods section.*
☒	☐	A description of all covariates tested
☒	☐	A description of any assumptions or corrections, such as tests of normality and adjustment for multiple comparisons
☐	☒	A full description of the statistical parameters including central tendency (e.g. means) or other basic estimates (e.g. regression coefficient) AND variation (e.g. standard deviation) or associated estimates of uncertainty (e.g. confidence intervals)
☐	☒	For null hypothesis testing, the test statistic (e.g. F, t, r) with confidence intervals, effect sizes, degrees of freedom and P value noted<br>*Give P values as exact values whenever suitable.*
☒	☐	For Bayesian analysis, information on the choice of priors and Markov chain Monte Carlo settings
☒	☐	For hierarchical and complex designs, identification of the appropriate level for tests and full reporting of outcomes
☒	☐	Estimates of effect sizes (e.g. Cohen's *d*, Pearson's *r*), indicating how they were calculated

Our web collection on statistics for biologists contains articles on many of the points above.

Software and code

Policy information about availability of computer code

Data collection	Amersham Imager 600 (GE Healthcare), LUMICKS C-trap instrument (Lumicks)
Data analysis	AIMLESS version 0.7.7 https://www.ccp4.ac.uk/; BUCCANEER version 1.6.11 https://www.ccp4.ac.uk/;CCP4i2 version 7.1.018 https://www.ccp4.ac.uk/;COOT version 0.9.6 https://www2.mrc-lmb.cam.ac.uk/personal/pemsley/coot/;DIALS version 3.dev.659-g0b5a5c991 https://dials.github.io/; Excel 2016 Microsoft RRID: SCR_016137;MolProbity version 4.4 http://molprobity.biochem.duke.edu/;PHASER version 2.8.3 https://phenix-online.org/;PyMOL Tversion 2 he PyMOL Molecular Graphics System https://pymol.org/2/;R version 3.2.4 R Foundation for Statistical Computing https://www.r-project.org/;REFMAC5 version 5.8.0403 https://www.ccp4.ac.uk/;XIA2 version 3.9.dev0 https://xia2.github.io/index.html;ColabFold v1.5.5 https://github.com/sokrypton/ColabFold;GraphPadPrism v10 GraphPad RRID: SCR_002798;ImageJ NIH RRID: SCR_003070;Thermo Xcalibur Qual Browser v. 4.2.47 ThermoFisher Cat# OPTON-30965; OriginPro v10.2 https://store.originlab.com/store/.

For manuscripts utilizing custom algorithms or software that are central to the research but not yet described in published literature, software must be made available to editors and reviewers. We strongly encourage code deposition in a community repository (e.g. GitHub). See the Nature Portfolio guidelines for submitting code & software for further information.

Data

Policy information about availability of data

All manuscripts must include a data availability statement. This statement should provide the following information, where applicable:

- Accession codes, unique identifiers, or web links for publicly available datasets
- A description of any restrictions on data availability
- For clinical datasets or third party data, please ensure that the statement adheres to our policy

The crystallographic structures of KorBdeltaN30deltaCTD and KorBdeltaN30deltaCTD-KorA-OA have been deposited in the PDB with accession codes: 8QA8 and 8QA9, respectively. The crystallographic structure of KorA-DNA complex (PDB code: 2W7N) was also used in this study. Deep sequencing data generated in this study have been deposited in the GEO database under the accession code GSE274567. All uncropped images and data presented in figures are available in Source Data.

Research involving human participants, their data, or biological material

Policy information about studies with human participants or human data. See also policy information about sex, gender (identity/presentation), and sexual orientation and race, ethnicity and racism.

Reporting on sex and gender	NA
Reporting on race, ethnicity, or other socially relevant groupings	NA
Population characteristics	NA
Recruitment	NA
Ethics oversight	NA

Note that full information on the approval of the study protocol must also be provided in the manuscript.

Field-specific reporting

Please select the one below that is the best fit for your research. If you are not sure, read the appropriate sections before making your selection.

☒ Life sciences ☐ Behavioural & social sciences ☐ Ecological, evolutionary & environmental sciences

For a reference copy of the document with all sections, see nature.com/documents/nr-reporting-summary-flat.pdf

Life sciences study design

All studies must disclose on these points even when the disclosure is negative.

Sample size	No sample size calculation was performed. For ITC experiment the number of replicate is 2, for all other experiment numbers of replicates are three or more (see relevant figure legend). The number if replicates is standard/common practice in the field.
Data exclusions	No data were excluded from the analyses
Replication	We routinely analyzed multiple independent strains, multiple different protein aliquots or purification to verify the phenotypes observed. All experiments were performed at least twice or more to ensure reproducibility, and similar results were obtained throughout.
Randomization	Strains for different experiments were selected randomly for inoculation from plate. All strains were grown under similar conditions, hence are equivalent at the start of the experiment. Observed differences are due to the difference in genotype of analyzed strains in this study.
Blinding	This was not necessary for in-bulk/population-averaged microbiology study. All strains were grown under similar conditions, hence are equivalent at the start of the experiment. Observed differences are due to the difference in genotype of analyzed strains in this study.

Reporting for specific materials, systems and methods

We require information from authors about some types of materials, experimental systems and methods used in many studies. Here, indicate whether each material, system or method listed is relevant to your study. If you are not sure if a list item applies to your research, read the appropriate section before selecting a response.

Materials & experimental systems

n/a	Involved in the study	
☐	☒	Antibodies
☒	☐	Eukaryotic cell lines
☒	☐	Palaeontology and archaeology
☒	☐	Animals and other organisms
☒	☐	Clinical data
☒	☐	Dual use research of concern
☒	☐	Plants

Methods

n/a	Involved in the study	
☒	☐	ChIP-seq
☒	☐	Flow cytometry
☒	☐	MRI-based neuroimaging

Antibodies

Antibodies used

polyclonal antibodies against KorB and KorA (custom synthesis, University of Birmingham, UK) (and further purified in the lab of Prof. Chris Thomas, University of Birmingham, UK) and Goat Anti-Rabbit IgG H&L (HRP) secondary antibody (ABCAM, UK cat#AB6721)

Validation

The specificity of primary antibodies against KorB and KorA used in this study was verified against lysates from deletion mutant strains or non-tagged strains of E. coli. Both antibodies have been used and reported in previous publications (see publications PMID: 7473715 and PMID: 10564465) from Prof. Chris Thomas' group at the University of Birmingham, UK. Secondary antibody: Goat Anti-Rabbit IgG H&L (HRP) secondary antibody is available commercially (ABCAM, UK cat#AB6721)

Plants

Seed stocks

Not relevant

Novel plant genotypes

Not relevant

Authentication

Not relevant
